# Apoptosis in the Extraosseous Calcification Process

**DOI:** 10.3390/cells10010131

**Published:** 2021-01-12

**Authors:** Federica Boraldi, Francesco Demetrio Lofaro, Daniela Quaglino

**Affiliations:** 1 Department of Life Sciences, University of Modena and Reggio Emilia, 41125 Modena, Italy; francescodemetrio.lofaro@unimore.it (F.D.L.); daniela.quaglino@unimore.it (D.Q.); 2 Interuniversity Consortium for Biotechnologies (CIB), Italy

**Keywords:** ectopic mineralization, extracellular matrix, cell death, apoptosis, vascular tissue, cartilage

## Abstract

Extraosseous calcification is a pathologic mineralization process occurring in soft connective tissues (e.g., skin, vessels, tendons, and cartilage). It can take place on a genetic basis or as a consequence of acquired chronic diseases. In this last case, the etiology is multifactorial, including both extra- and intracellular mechanisms, such as the formation of membrane vesicles (e.g., matrix vesicles and apoptotic bodies), mitochondrial alterations, and oxidative stress. This review is an overview of extraosseous calcification mechanisms focusing on the relationships between apoptosis and mineralization in cartilage and vascular tissues, as these are the two tissues mostly affected by a number of age-related diseases having a progressively increased impact in Western Countries.

Apoptosis is a well-known type of programmed cell death normally occurring, either during development or aging, as a homeostatic mechanism controlling the cellular component within tissues by removing damaged or unnecessary cells under a wide variety of stimuli and conditions [1]. Dysregulation of apoptotic signaling and inappropriate apoptosis have been involved in the occurrence and progression of many diseases and of their complications, including ectopic calcification [2,3].

More details on signals and on mechanisms that can induce apoptosis or, if removed prior to complete cell death, can reverse cell fate, are beyond the purpose of the present work (for extended reviews, see for instance [4,5,6]).

The calcification process is actively regulated by cells responsible for the formation, organization, and maintenance of the extracellular matrix and contributes to the deposition and accumulation of inorganic moieties (Ca^2+^, Pi, Mg^2+^, CO_3_). Mineralization is physiologically restricted to bones, teeth, growth plate, and deep layers of the articular cartilage under the coordinated action of inhibitory and stimulatory factors [7,8]. In pathologic conditions, mineralization also occurs in soft connective tissues. Extraosseous or ectopic calcification can be due to genetic mutations (e.g., Pseudoxanthoma elasticum, generalized arterial calcification of infancy, Familial chondrocalcinosis, and Familiar tumoral calcinosis) [9,10] or can be the consequence of acquired chronic diseases (e.g., atherosclerosis, diabetes, chronic kidney disease, and osteoarthritis). Tissues mainly affected by aberrant mineralization are the cardiovascular system (i.e., vessels and valves) and the joints with diseases and clinical complications, which are the main cause of morbidity and mortality in the Western World and in the aging population, where a pro-osteogenic environment progressively takes place [11,12,13,14].

This review aims to provide an overview of extraosseous calcification mechanisms focusing on the relationships between apoptosis and mineralization in cartilage and vascular tissues.

## 1. Extraosseous Calcification

In physiological conditions, calcium (Ca^2+^) and inorganic phosphate (Pi) exceed their solubility in most tissues; however, calcification does not occur in soft connective tissues for the presence of circulating and local inhibitors [15]. When these regulatory mechanisms are skimpy, pathological mineralization overwhelms and takes place through active cell-mediated processes partially overlapping those observed during skeletal formation.

Although ectopic calcification can be due to mutation/s in a single or in few genes [9,10], in most cases, such as in aging and in several chronic degenerative disorders (e.g., atherosclerosis, diabetes, chronic kidney disease, and osteoarthritis), pathologic mineralization is multifactorial and involves a number of protein interactions and dysregulated signaling pathways [16,17]. On the basis of the pathogenic mechanism(s), extraosseous calcification is described as: (i) dystrophic, occurring in damaged tissues when serum calcium and phosphate are within a normal range [18]; (ii) metastatic, due to abnormal calcium and/or phosphate metabolism leading to hypercalcemia and/or hyperphosphatemia [18]; (iii) heterotopic ossification characterized by true bone tissue formed outside of the skeleton, as after surgery or traumatic injury [19]; (iv) calciphylaxis, often associated to end-stage kidney disease and characterized by ischemic necrosis of the skin and calcification of the tunica media and fibrosis of the intima of cutaneous arterioles associated with thrombotic occlusion [20].

In hard tissues, minerals are mainly deposited as hydroxyapatite, in contrast, in soft connective tissues, crystals of different compositions have been described (Table 1).

There are several patho-mechanisms responsible for ectopic calcification, as altered hormonal homeostasis [47], dysregulated angiogenesis and/or vascular repair mechanisms [48], and abnormal extracellular nucleotide metabolism [49,50]. More recently, a number of investigations have focused on the release of membrane vesicles, on the role of modified mitochondria-related pathways, and on the influence of oxidative stress on the occurrence and progression of soft connective tissue mineralization, as detailed in the following sections of the present review.

### 1.1. Membrane Vesicles

Membrane vesicles (also known as extracellular vesicles) are phospholipid-enclosed nanoparticles (30–2000 nm), that, based on their diameter and biogenesis, are comprised of: (i) exosomes (30–150 nm) of endosomal origin; (ii) matrix vesicles (or microvesicles, 50–1000 nm) generated by blebbing of the plasma membrane; (iii) apoptotic bodies (500–2000 nm) released from dying cells [51,52].

**Figure 1:**
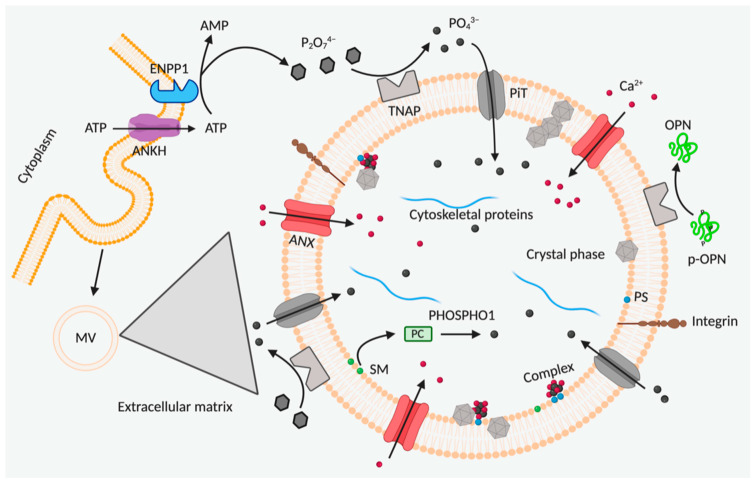
**MVs.** Mineral deposition is initiated by accumulation of calcium (Ca^2+^) and inorganic phosphate (PO_4_^3^^−^) into matrix vesicles (MVs) (for more details see the text) Abbreviations: ANKH = progressive ankylosis protein homolog, ANX = annexin; ENPP1 = nucleotide pyrophosphatase phosphodiesterase; OPN = osteopontin; PC = phosphocholine; PiT = phosphate transporter; P_2_O_7_^4^^−^ = pyrophosphate; PS = phosphatidylserine; TNAP = tissue non-specific alkaline phosphatase; SM = sphingomyelin.

Matrix vesicles (MVs) are small spherical bodies of cellular origin found in association with crystal deposits. However, MVs’ biogenesis is still under investigation as they could have a different origin depending on the cellular type as well as on the chemical and osmotic characteristics of the extracellular matrix. Some authors have identified vascular smooth muscle cells-derived MVs as exosomes [53], whereas other studies, performed on chondrocytes and osteoblasts [54,55,56,57], indicate that MVs originate from the cellular plasma membrane (Figure 1). MVs have been found in soft connective tissues (e.g., in calcific valvular stenosis, atherosclerosis) with structure/composition similar to skeletal MVs [57,58,59], suggesting that the mechanisms of extraosseous calcification are similar to those observed in normal skeletal development [60].

As initiators of calcification, MVs contain either regulators of phosphate homeostasis, or proteins and lipids serving as nucleation sites for crystal deposition [56]. By proteomic analysis it has been demonstrated that MVs contain several enzymes (tissue non-specific alkaline phosphatase (*ALP*/TNAP), nucleotide pyrophosphatase phosphodiesterase (*NPP1*/ENPP1), phosphoethanolamine/phosphocholine phosphatase (PHOSPHO-1), Na+/K+ ATPase, metalloproteinases (MMP-2, -3, and -13)], transport proteins (annexins (ANXs) as ANX II, V, and VI); sodium-dependent inorganic phosphate transporters (PiT-1, -2) and other proteins (integrins) and lipids (i.e., phosphatidylserine and phosphatidylethanolamine) [61]. MVs enzymes (TNAP and ENPP1), together with the cell associated progressive ankylosis protein homolog (ANKH), actively contribute to ectopic calcification. In particular, ANKH has been reported to regulate levels of extracellular and intracellular inorganic phosphate (Pi), transporting pyrophosphate (PPi), a strong inhibitor of soft connective tissue calcification [62,63] or, according to other studies, transporting ATP [64,65] that can be later converted to PPi by ENPP1. This last enzyme converts extracellular nucleoside triphosphates to AMP and PPi, whereas TNAP creates a pro-osteogenic environment by: (1) hydrolyzing PPi and enhancing extracellular inorganic phosphate (Pi) concentration, a mineralization promoter; and (2) modulating the phosphorylation of the non-collagenous bone protein osteopontin (OPN), that, in its phosphorylated status, inhibits hydroxyapatite formation and growth [66]. Pi levels into MVs are governed both by the Pi influx through PiT-1 and by the Pi generated from MVs membrane lipids (i.e., phosphocholine, phosphoethanolamine). These lipids are engendered from the membranes through the action of phospholipases and then release intra-vesicular Pi upon hydrolysis by PHOSPHO-1 [67].

Ca^2+^ influx into MVs occurs through ANX channels. The high concentration of calcium and phosphate into MVs triggers their precipitation, followed by mineral stabilization due to the binding of crystals to phosphatidylserine, an anionic phospholipid with high affinity for Ca^2+^. Initially, these complexes are made of amorphous calcium phosphate, which is progressively transformed into hydroxyapatite [68]. Crystals, along their growth, can destroy MVs’ membranes and are released into the extracellular matrix, where they interact with Ca^2+^ and PO_3_^−4^ moieties continuing their growth [56,61,69].

Annexins are expressed in many different cell types and exert many functions, including exocytosis, membrane fusion, ion channels, and receptors for extracellular matrix proteins (e.g., collagens type I, II, and X and proteoglycans). In particular, ANX II, V and VI, not only mediate Ca^2+^ influx into MVs, but they form Ca^2+^ channels also in hypertrophic growth plate chondrocytes leading to the influx of Ca^2+^, stimulation of terminal differentiation, including up-regulation of specific genes, the release of MVs, induction of matrix mineralization and apoptosis [70].

Similarly to MVs, apoptotic bodies (ABs) can be the initiators of the mineralization process [71,72,73]. However, ABs, if compared to MVs, exhibit some differences in size, structure and composition [74]. In particular, ABs do not contain or contain only a few ANX II, V, and VI, which, in this context, are not required for calcification. It has been shown that, after blocking ANX channel activities with specific antibodies, ABs accumulate calcium on their outer membrane surface [73], where phosphatidylserine is exposed, allowing calcium binding. Therefore, ABs and MVs use different mechanisms to induce calcification: the first mineral phase occurs inside MVs, whereas it takes place on the outer membrane surface in ABs [16,74].

### 1.2. Mitochondria and Oxidative Stress

Ultrastructural observations, performed already in the 1970s, demonstrated the presence of hydroxyapatite deposition within mitochondria in bone as well as in soft connective tissue, thus highlighting the relationship between these organelles and the calcification process [75]. Mitochondria are key players in cellular energy metabolism, produce adenosine triphosphate (ATP) and a number of biosynthetic intermediates, participate in the redox balance and are involved in autophagy and apoptosis [76] and may also be regarded as initiators of intracellular calcification [77]. Within mitochondria, several mineral inclusions can be found in the form of needle-shaped crystals or of fine granules [77,78,79]. The former start to be deposited close to the cristae and later spread to the whole mitochondrial matrix, whereas the latter begin to develop in the mitochondrial matrix and thereafter grow close to cristae [77]. To be noted that dense granules of calcium-phosphate have been observed within mitochondria of many different cell types, thus indicating that these findings are not peculiar for a specific cell type [80,81,82]. Mitochondria initiate calcification through the interaction of phosphatase enzymes, including alkaline phosphatase, with calcium-binding phospholipids forming Ca- and P-rich electron-dense granules, which are released from cells to mineral nucleation sites in the extracellular space [83,84].

More recent investigations revealed that mitochondria further contribute to ectopic calcification producing reactive oxygen species (ROS), thus causing oxidative stress damage and promoting mitochondria-mediated apoptosis and subsequent calcification. Within cells, mitochondria undergo continuous changes alternating fusion and fission events [85], fission events being associated with calcification. In particular, dynamin-related protein 1 is the main player in mitochondrial fission, and its activity is regulated by different post-translational modifications (e.g., phosphorylation, ubiquitination, and S-nitrosylation) [86].

Suppression of mitochondrial fission reduces apoptosis, ROS, runt-related transcription factor 2 (Runx2) protein expression, and calcium deposition, thus inhibiting extraosseous mineralization [87].

**Figure 2:**
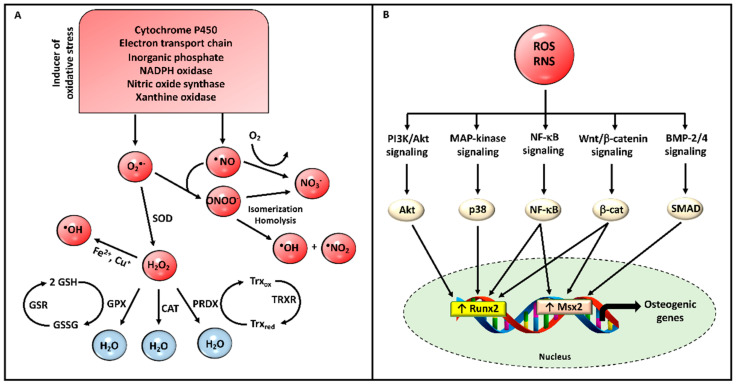
(**A**) Generation of ROS and RNS. When ROS and RNS production exceeds the capacity of antioxidant systems, an oxidative stress condition is created, which causes damage to the macromolecules (i.e., nucleic acids, proteins, carbohydrates, and lipids). (**B**) Signal transduction pathways regulated by ROS and RNS. The activation of these pathways induces nuclear translocation of transcription factors, which lead to the activation of osteogenic genes. Abbreviations: ROS = reactive oxygen species; RNS = reactive nitrogen species; BMP-2/4 = bone morphogenetic protein-2 and -4, CAT = catalase, GPX = glutathione peroxidase, GSH = reduced glutathione, GSR = glutathione disulfide reductase, GSSG = glutathione disulfide, H_2_O_2_ = hydrogen peroxide, O_2_^●−^ = superoxide anion, ^●^OH = hydroxyl radical, ONOO^−^ = radical peroxynitrite, Msx2 = muscle segment homeobox 2, ^●^NO = nitric oxide, ^●^NO_2_ = nitrogen dioxide radical; NO_3_^−^ = nitrate; NF-κB = nuclear factor kappa-light-chain-enhancer of activated B cells, PI3K/Akt = phosphatidylinositol 3-kinase/protein kinase-B, PRDX = peroxiredoxin, Runx2 = runt-related transcription factor 2, SOD = superoxide dismutase, Trx_red_ = reduced thioredoxin, Trx_ox_ = oxidized thioredoxin, TrxR = thioredoxin reductase.

The source of ROS is not limited to mitochondria. Together with reactive nitrogen species (RNS), ROS are produced in various cell compartments (cell membrane, cytoplasm, endoplasmic reticulum, and peroxisomes) by enzymes such as nicotinamide adenine dinucleotide phosphate oxidases, nitric oxide synthase (NOS), xanthine oxidase, cytochrome P450 and cyclo-oxygenase [88] (Figure 2A). The redox signaling mechanisms activated by hydrogen peroxide (H_2_O_2_) and radical peroxynitrite (ONOO^−^) are similar, although ONOO^−^ has higher reactivity. In particular, ONOO^−^, once protonated, can be: (i) activated by homolysis forming hydroxyl radical plus nitrogen dioxide radical; (ii) degraded by isomerization to nitrate (Figure 2A). Under physiological conditions, ROS and RNS production is tightly regulated by antioxidant enzymes; however, when their formation exceeds the antioxidant capacity of cells and tissues, an oxidative stress condition takes place. Even though ROS/RNS are known to be deleterious for cells, they also represent important and necessary signaling molecules [89,90]. Therefore, the balance between oxidant/antioxidant molecules must be finely tuned to avoid uncontrolled signaling mechanisms as demonstrated, for instance, by nuclear factor kappa-light-chain-enhancer of activated B cells (NF-κB), which is up- or down-regulated by ROS/RNS in concentration- and cell-dependent manners [91,92].

Mitochondrial ROS and RNS have been shown to activate NF-κB and NF-κB-dependent osteo-inductive signaling pathways (Figure 2B) [91,93]. NF-κB is located in the cytoplasm in a latent form that binds to several inhibitory proteins (IκBs) and can be activated by classical or alternative pathways [92]. Therefore, in response to several stimuli, IκB is phosphorylated, leading to its ubiquitination and proteasomal degradation. Subsequently, NF-κB is free to translocate into the nucleus and to bind to NF-κB-responsive elements, up- or down-regulating the expression of osteogenic factors (e.g., *ALP*) and anti-mineralization proteins (e.g., ANKH), respectively [93]. Several studies, performed in in vitro and in vivo calcification models, have highlighted that inhibition/suppression of NF-κB can reduce mineralization [93,94,95]. Moreover, NF-κB inhibits glycogen synthase kinase-3β determining the activation of the canonical Wnt pathway that can also be directly activated by ROS and RNS [96,97], controlling numerous cellular processes, as osteogenic trans-differentiation [98] and calcification [99].

Oxidative stress up-regulates the expression of Runx2 through the activation of the phosphatidylinositol 3-kinase/protein kinase-B/Runx2 signaling pathway (PI3K/AKT/Runx2) or the p38 MAP kinase signaling pathway [91,99] leading to osteogenic trans-differentiation and promoting calcification [100,101] (Figure 2B). Moreover, ROS/RNS up-regulate bone morphogenetic protein-2 and -4 (BMP2 and BMP4) expression [102,103,104], which, through SMADs, act on several transcription factors such as Runx2, muscle segment homeobox 2 (Mxs2), and osterix, essential for pro-osteoblastic differentiation (Figure 2B) [105]. Consistently, Runx2 induces the expression of bone matrix proteins such as collagen type I, osteocalcin (OC), OPN, bone sialoprotein, and TNAP activity [106,107].

## 2. Apoptosis and Cartilage Calcification

Articular cartilage is typically composed of chondrocytes embedded within a rather amorphous extracellular matrix (ECM) comprised of collagen (mainly type II), different proteoglycans (e.g., perlecan, aggrecan), structural proteins involved in cell-matrix and matrix-matrix interactions (e.g., cartilage matrix protein, fibronectin), proteinases and their inhibitors [108].

Endochondral ossification, a physiologic mechanism occurring during skeletal development, is essential in growth plates of developing long bones [109], in the antlers of deers [110], and at the tendon and ligament insertions into bones [111]. It is a complex multistep process orchestrated by chondrocytes undergoing a sequence of events (proliferation, hypertrophy, terminal differentiation, and cell death), and it is restricted to regions of terminally differentiated chondrocytes (i.e., areas close to chondro-osseous junctions) [112]. In particular, hypertrophic chondrocytes are involved in the regulation of cartilage remodeling and calcification as well as in the bone vascularization process, producing collagen type X and activating osteoblast-related genes (e.g., MMP-13, ALP, OPN, bone sialoprotein, osterix, OC, and Runx2) [113]. These cells undergo osteogenic trans-differentiation releasing MVs into the ECM and initiating the calcification process [114,115,116].

Changes in these pathways may lead to pathologic conditions, as in osteoarthritis (OA), the most common degenerative joint disease in older individuals, which can be considered a paradigmatic model of aberrant cartilage calcification due to unbalanced cartilage homeostasis due to excessive catabolic activity. During OA progression, chondrocytes undergo important phenotypic changes and exhibit an abnormally increased rate of maturation [117]. Hypertrophic chondrocytes, as observed in the growth plate, acquire a more catabolic phenotype increasing the secretion of degradative enzymes such as MMP-13 and a disintegrin and metalloproteinase with thrombospondin motifs (ADAMTS)-4 and -5, which destroy the ECM. Degradation of cartilage ECM proteins (e.g., collagen and fibronectin), secretion of damage-associated molecular patterns (DAMPs) and alarmins (e.g., calgranulins S100A8 and S100A9) can induce an inflammatory response [118] with the release of cytokines, NO and ROS, which, in turn, increase chondrocyte catabolism. Therefore, chondrocyte catabolism, tissue degradation, and inflammation contribute to the severity of OA [119].

The observation that in OA there are numerous empty lacunae and hypocellularity have suggested that chondrocyte cell death can participate in the pathogenesis of OA [72,120,121]. Consistently, in OA increased chondrocyte death was detected compared to healthy cartilage [122,123,124], and both apoptotic and necrotic mechanisms have been described [120,125,126,127]. Roach et al. [128] coined the term “chondroptosis”, to indicate a specific form of death, in which cells are characterized by shrunk and condensed chromatin, increased rough endoplasmic reticulum and Golgi apparatus, and the presence of autophagic vacuoles. Interestingly, during the early stages of OA, the superficial layer and the middle zone of cartilage are characterized by increased death of chondrocyte due to a combination of apoptosis and autophagy, the latter as an adaptative mechanism activated in sublethal conditions that may lead to cell death after a point of no return [127,129]. By contrast, in the deeper zone, in both human and animal OA models, apoptotic cell death was revealed associated with abnormal cartilage calcification [129,130].

**Figure 3:**
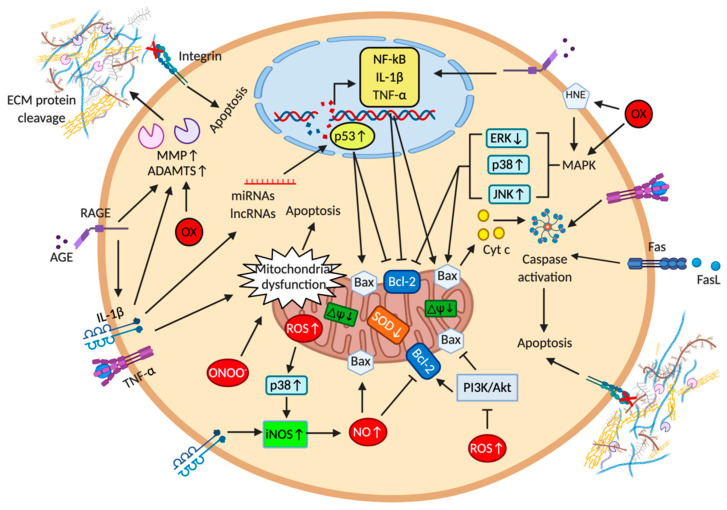
Schematic representation of extrinsic and intrinsic factors involved in chondrocyte apoptosis. Abbreviations: ADAMTS = disintegrin and metalloproteinase with thrombospondin motifs; AGE = advanced glycation end products; Bax = Bcl-2-associated X; Bcl-2 = B-cell lymphoma protein 2; Cyt c = cytochrome c; ΔΨ = mitochondrial membrane potential, ECM = extracellular matrix; ERK = extracellular-signal-regulated kinase; FasL = Fas ligand; HNE = 4-hydroxynonenal; IL-1β = interleukin 1 beta; iNOS = inducible nitric oxide synthases; JNK = c-Jun N-terminal kinases; LncRNAs = long non-coding RNA; MAPK = mitogen-activated protein kinase; miRNAs = microribonucleic acids; MMP = matrix metalloproteinases; NF-κB = nuclear factor kappa-light-chain-enhancer of activated B cells; NO = nitric oxide; ONOO^−^ = radical peroxynitrite, OX = oxidative stress; PI3K/Akt = phosphatidylinositol 3-kinase/protein kinase-B; RAGE = receptor for advanced glycation end products, ROS = reactive oxygen species; SOD = superoxide dismutase; TNF-α = tumor necrosis factor alpha.

According to some authors, a positive correlation exists between apoptosis and severity of cartilage damage [120,121,126,127,131] and different molecular signals (Figure 3) can induce chondrocyte apoptosis, even if it has not yet been clarified whether apoptosis is the final step in the terminal differentiation of articular chondrocytes or whether it occurs independently [124]. As a consequence of chondrocyte apoptosis, there is the formation of ABs, which, remaining in the lacunae of chondrocytes, release their content (e.g., nucleotide pyrophosphohydrolase and alkaline phosphatase) [72] and may trigger the mineralization process.

It is worth mentioning that cartilage is avascular, and therefore, the inflammatory-derived degradative process is related to persistent infiltration of leucocytes from surrounding tissues and to the presence of cytokines and mediators in the synovial fluid. Interestingly, one study showed that, in the early stages of OA, some chondrocytes, isolated from degenerated rat cartilage and expressing type II collagen+/CD163+, had phagocytic activity and, therefore, might have a role in the clearance of dead chondrocytes. However, these cells disappear during OA progression [132].

Further studies have supported the finding that in addition to chondrocyte-derived ABs, also MVs released by hypertrophic chondrocytes participate in pathological cartilage mineralization [30,56,72,133,134]. For example, ABs isolated from cultured chondrocytes treated with NO or anti-Fas, as inducers of chondrocyte apoptosis [135,136], are capable of promoting in vitro mineralization [72]. A study performed on knee articular cartilage from OA and control samples showed, in patients, the co-presence of chondrocyte apoptosis and of hydroxyapatite (HA) microcrystals, highlighting the connection between apoptosis and ectopic mineralization [126].

In in vitro models, in the absence of endogenous pro-osteogenic stimuli, addition to the culture medium of Pi or β-glycerophosphate is required to induce and/or stimulate the calcification process [137,138,139,140] as demonstrated in ATDC5 chondrocytes, where Pi supplementation accelerates terminal chondrocyte differentiation and induces apoptosis-dependent mineralization. Moreover, the ability of a caspase inhibitor to reduce crystal formation indicated that calcification was, at least partially, dependent on caspase activity [141]. Recently, it has been shown that, under oxidative stress, ABs derived from endplate chondrocytes up-regulated the expression of osteocalcin, RUNX2, and ALP and down-regulated ENPP1 and ANK expression, implying that oxidative stress and ABs alter PPi metabolism, increasing Pi content [142].

Interestingly, it has been observed that calcium-phosphate crystals (e.g., OCP and HA) exerted an amplifying role in chondrocyte apoptosis [135,143]. In particular, chondrocytes were capable of engulfing crystals, which were dissolved into lysosomes determining an increase of Ca^2+^ intracellular levels [144,145], this ion being a positive regulator of chondrocyte differentiation, apoptosis, and mineralization [146]. Moreover, calcium-phosphate crystals are closely associated with cartilage degradation because they up-regulate the expression of cytokines (e.g., IL-6) and of cartilage-degrading enzymes (e.g., MMP-13 and collagenase), thus condition cell fate and behavior [147,148].

### 2.1. Extracellular Matrix Components in the Context of Cartilage Apoptosis

Studies performed on human and animal chondrocytes have demonstrated that changes in matrix components can trigger chondrocytes cell death and that several risk factors (e.g., traumatic joint injury, physical activity, genetics, age, and obesity) are related to increased catabolic processes that through pro-inflammatory cytokines, degradative enzymes, loss, and breakdown of matrix constituents sustain the perpetuation of a catabolic cycle starting from the superficial zone and proceeding towards the deeper zone [149,150]. In particular, in OA cartilage, it has been observed a disruption, driven by MMP-13 and ADAMTS-4 and -5, of the network comprised of collagen type II, aggrecan, fibronectin, hyaluronan, and other non-collagenous matrix proteins [151], determining the formation of ECM protein fragments [152,153,154] which induce an inflammatory response, an increase of catabolic proteases (e.g., collagenases, aggrecanases, hyaluronidases) and induction of apoptosis (Figure 3). Moreover, age-related accumulation of advanced glycation end products (AGE) may further contribute to cartilage damage [155,156] as demonstrated in OA cartilage by Loeser and collaborators [157], showing increased expression of AGE receptors (RAGE) were associated with activated inflammatory pathways and increased MMP-13 production.

Because the cartilaginous ECM plays a crucial role in regulating cell growth, differentiation, adhesion, and survival, degeneration and loss of cartilage matrix can modify the ability of chondrocytes to adhere to ECM. For instance, cleaved collagen type II can induce, in vitro, a significant increase of chondrocyte apoptosis [158]. Consistently, it has been demonstrated that integrins, transmembrane receptors involved in cell-matrix interactions, can mediate cell survival, preventing apoptosis [159], as demonstrated in chondrocytes cultured on fibronectin, which underwent apoptosis when anti-α5β1 integrin antibodies were added to the medium [160]. Similarly, in OA animal models, changes in the expression and localization of α2, α4, and α5 integrins were observed [161], and increased expression of MMP-2 and MMP-3 associated with cell reduction and increased apoptosis compared to wild-type (WT) mice have been detected in the cartilage of α1-KO mice [162].

Altogether, these data indicate that altered cartilage matrix composition and organization and chondrocyte apoptosis can generate a feedback loop where the progression of one worsens the other.

### 2.2. Death Receptors and Cytokines in the Context of Cartilage Apoptosis

Apoptosis is typically triggered by the interactions between Fas and Fas ligand (FasL), members of the tumor necrosis factor (TNF)-receptor and TNF family, respectively (Figure 3). Fas is a transmembrane protein containing a cytoplasmic death domain essential for the induction of apoptosis, therefore binding between FasL and Fas determines the recruitment of the adaptor protein Fas-associated death domain, which recruits caspase-8 and caspase-10 to form the death-inducing signaling complex. Caspase-8, in turn, induces apoptosis by two pathways: (i) activating caspase-3 or (ii) cleaving BH3 interacting death domain agonist and consequently inducing mitochondrial dysfunction with cytochrome-c release and caspases-9 and -3 activation.

It has been demonstrated that, in the early stages of OA, hypocellularity is associated with enhanced levels of Fas, FasL, and caspase-8 [163,164]. Consistently, chondrocytes from OA damaged areas have higher Fas expression than those from non-lesioned areas [165]. Interestingly, in human and in mouse OA cartilage, a correlation was found between apoptosis and the transcription factor hypoxia-inducible factor (HIF)-2α through enhanced Fas expression [166,167].

Moreover, several in vitro and in vivo studies have demonstrated that pro-inflammatory cytokines, such as TNF-α and IL-1β, are produced by chondrocytes and play a key role in OA pathogenesis [168]. These cytokines: (i) induce the over-expression of MMP-1, -3, -13 and ADAMTS-5, favoring proteolysis and cartilage ECM loss [169,170]; (ii) prevent the normal production of collagen type II and aggrecan [170,171]; (iii) stimulate NO and cyclooxygenase-2 production contributing, indirectly, to chondrocyte cell-death [172]; (iv) induce autocrine production of IL-1β and TNF-α [173]; and (v) cause mitochondrial dysfunction, reducing mitochondrial DNA (mtDNA) integrity and mtDNA repair capacity, which correlates with increased chondrocyte apoptosis [174,175]. Finally, it has been demonstrated that TNF-α-induced chondrocytes apoptosis is dependent on the balance between survival (i.e., ERK1/2) and death (i.e., p38 MAPK and JNK) signaling pathways [176,177].

### 2.3. Mitochondria and Reactive Oxygen and Nitrogen Species in the Context of Cartilage Apoptosis

Mitochondria play a crucial role in cellular function and survival, and the alterations in mitochondrial structure and function (e.g., increase in membrane permeability, depolarization, decrease in ATP production, and the release of cytochrome c) precede the classic signs of cell death [178,179]. Compared to normal chondrocytes, those from OA cartilage show diminished efficiency of electron transfer pathways associated with mitochondrial membrane depolarization, which led to apoptosis following caspase-9 activation and release of cytochrome c from the inner mitochondrial membrane to the cytosol [178,180]. In addition, proteomic analysis showed in OA chondrocytes higher ROS levels [181] and a significant decrease of manganese-superoxide dismutase (Mn-SOD), a mitochondrial antioxidant enzyme (Figure 3). The treatment of chondrocytes with antioxidants, preventing oxidative stress- derived mitochondrial damage, efficiently reduced apoptosis [182].

Interestingly, Ca^2+^ influx is important in apoptosis as it is able to induce ROS generation, mitochondrial depolarization, and mtDNA damage [183]. Transient receptor potential ankyrin 1 (TRPA1), a membrane-associated cationic channel, can be activated by RNS and ROS [184], causing an influx of cation ions, in particular of Ca^2+^. TRPA1 expression is up-regulated in primary human OA chondrocytes by inflammatory cytokines (e.g., IL-1β, TNF-α) and favors the production of MMP-1, MMP-3, MMP-13, IL-6, and prostaglandin E2 [185,186]. Furthermore, in an OA experimental model, Yin and collaborators [187] demonstrated that IL-1β increases TRPA1 expression contributing to overload Ca^2+^ influx, which, in turn, causes a significant reduction of mitochondrial membrane potential, activating the intrinsic apoptotic pathway.

Several in vitro and in vivo studies provided clear evidence that excessive generation of ROS and of RNS in OA chondrocytes induces altered gene transcription and protein synthesis and that in this setting, apoptosis is activated [120,126,188]. For instance, in the cartilage endplate, intracellular production of H_2_O_2_ induced apoptosis and calcification via ERK/p38/p65 signaling pathway [189]. Moreover, H_2_O_2_ generated by the hypoxanthine-xanthine oxidase system causes higher levels of lipid peroxidation, cartilage ECM degradation, and apoptosis by inducing caspase activation, down- and up-regulating B-cell lymphoma 2 (Bcl-2) and Bcl-2 Associated X (Bax) expression, respectively, and suppressing Akt kinase activity [190]. Similarly, H_2_O_2_-treated chondrocyte apoptosis has been observed as the result of altered expression of inducible NOS (iNOS), PI3K/Akt, caspase-9, and caspase-3 protein expression levels [191] or through Ca^2+^ signaling due to Ca^2+^ release from intracellular storage and activation of caspase-3 [192], thus underlining that the heterogeneity of apoptotic signaling pathways is context-dependent.

NOS can form NO from O_2_ and L-arginine. There are three isoforms of NOS: two constitutive (i.e., endothelial and neuronal NOS) and one inducible (i.e., iNOS). In normal conditions, chondrocytes express low levels of iNOS, but in human OA cartilage iNOS expression is induced by different stimuli (e.g., abnormal biomechanical forces and pro-inflammatory cytokines) [193,194]. An increase of NO production has been observed in OA cartilage leading to enhanced MMPs activity and inhibition of proteoglycan synthesis [193]. Interestingly, NO, in its nitrosonium form (NO^+^), can bind to cysteine residues to form S-nitrosothiols, which, in turn, regulate transcription factor and enzyme activities such as NF-κB, activator protein-1 (AP-1), p21 kinase, c-Jun N-terminal kinases (JNKs), caspase-3, and caspase-9 [195]. Whereas some studies showed that NO donors given to cultured chondrocytes caused apoptosis [135,196], other published results reported that the overexpression of the iNOS gene in transfected chondrocytes did not determine cell death. Therefore, it has been suggested that NO, by itself, can be protective under certain oxidative stress conditions and that cell death occurs only when other ROS species are involved [197,198]. NO rapidly reacts with O_2_^●−^ forming the free radical peroxynitrite (ONOO^−^), which induces mitochondrial dysfunction in chondrocytes leading to caspase-independent apoptosis through Ca^2+^-dependent protease activation (i.e., calpain) [199].

### 2.4. Microribonucleic Acids and Long Non-Coding RNA in the Context of Cartilage Apoptosis

Microribonucleic acids (miRNAs or miRs) are small nucleotide non-coding RNAs, whose principal function is to regulate gene expression at transcriptional and post-transcriptional levels binding to specific sequences of target mRNAs.

Chondrocytes express many miRNAs, which, governing hundreds of other genes, regulate cartilage development, homeostasis, and may lead to pathologic conditions [200]. Moreover, several studies have highlighted that aberrant expression of some miRNAs promotes chondrocyte apoptosis (Figure 3), and given the relationship between apoptosis and ectopic mineralization, these miRNAs may also have an effect on HA deposition.

In OA chondrocytes it has been observed a number of up-regulated miRNAs. In particular, miR-9 down-regulates monocyte chemoattractant protein-induced protein 1 expression, promoting IL-6 expression and chondrocyte apoptosis [201]; miR-103 induces cell apoptosis and suppresses cell proliferation down-regulating PI3K/AKT pathway by blocking sphingosine kinase 1 expression [202]; miR-34a promotes apoptosis by directly regulating the silent information regulator 1 (SIRT1)/p53 signaling pathway [203] or through the delta-like protein 1 via PI3K/AKT pathway [204]. SIRT1, in particular, is a deacetylase playing a crucial role in the prevention of apoptosis, enhancing acetylated p53 and Bax and down-regulating Bcl-2. Silencing miR-34a by locked-nucleotide-analog (LNA)-modified-anti-sense can decrease IL-1β-induced rat chondrocyte apoptosis, affecting collagen type II expression and iNOS activity [204]. Furthermore, as shown in in vitro (i.e., lipopolysaccharide treated chondrocytes) and in vivo (rat OA model) experiments, miR-363-3p controls apoptosis enhancing p53 and caspase-3 expression, whereas it down-regulates nuclear factor-erythroid 2-related factor 1 gene expression, which serves as a promoter of nuclear and mitochondrial interactions, modulating essential processes ranging from protein production to mitochondrial biogenesis [205]. Recently, in a rat OA model, it has been demonstrated that miR-495 enhances chondrocyte apoptosis and senescence acting through AKT1 and the AKT/mTOR signaling pathways. Moreover, up- or down-regulated expression of miR-495 stimulated or inhibited, respectively, the expression of ECM-related enzymes, such as MMP-1, MMP-13, and ADAMTS-5 [206]. Inhibiting miR-495 can suppress chondrocyte apoptosis [207].

By contrast, chondrocyte apoptosis is associated with the down-regulation of miR-29a, which favors Bax expression leading to caspase-3 activation [208].

In addition to studies disclosing the involvement of an increased number of miRs in regulating chondrocyte apoptosis (e.g., miR-10a-5p, miR-139, and miR-146) [209,210,211], in the last years, great attention has also been paid to long non-coding RNAs (lncRNAs), non-protein-coding transcripts characterized by a minimal length of 200 nucleotides, which govern key cellular processes (e.g., apoptosis and differentiation). It has been reported that a number of lncRNAs were involved in cartilage diseases contributing, for example, to ECM degradation, apoptosis, and osteogenic differentiation [212,213,214,215]. For instance, growth arrest-specific 5 (GAS5), an RNA gene affiliated with the lncRNA class, was up-regulated in OA, acting as a negative regulator of miR-21, causing an increased expression of different MMPs (e.g., MMP-2, MMP-13, and ADAMTS-4) and favoring apoptosis [216]. Another lncRNA involved in promoting the expression of the MMP family and chondrocyte apoptosis is the HOX antisense intergenic RNA (HOTAIR), which is significantly up-regulated in OA compared to normal cartilage [217]. In an in vitro OA model (i.e., IL-1β-stimulated rabbit primary chondrocytes), HOTAIR is increased, whereas HOTAIR knockdown reduces IL-1β-induced apoptosis [218].

## 3. Apoptosis and Vascular Calcification

In vascular tissue, apoptosis of endothelial (EC) and vascular smooth muscle cells (VSMCs) represents a physiologic process that, during developmental stages, allows programmed capillary regression [219] and ductus arteriosus formation [220], and, during the postnatal period, regulates blood vessels’ remodeling [221]. However, apoptosis also plays a key role in many vascular and vascular-related diseases (e.g., atherosclerosis and hypertension) and can significantly contribute to vascular calcification (VC) [222,223,224]. In vitro studies have demonstrated that homocysteine, a known predictive factor of vascular diseases, can induce endothelial cell apoptosis through increased generation of reactive oxygen species. Moreover, homocysteine can promote vascular calcification by increasing lipid peroxidation. In the light of these data, it was suggested that lowering plasma homocysteine levels can ameliorate endothelial homeostasis and vascular calcification [225].

VC is a typical and life-threatening complication observed in many diseases, including atherosclerosis, diabetes, coronary artery disease, and end-stage renal disease under the influence of inflammatory cytokines and growth factors [226]. It is characterized by the pathological deposition of minerals (e.g., HA) in vessel walls and in cardiac and vascular valves, thus altering tissue architecture and function as well as cell behavior [14]. Moreover, calcification is typically associated with a VSMC shift from a contractile to an osteogenic phenotype, contributing to altering the local balance between pro- and anti-calcifying factors and favoring the release of matrix vesicles [227]. The phenotypic transition of VSMCs is characterized by a down-regulation of smooth muscle 22-α protein, alpha-smooth muscle actin, and smooth muscle myosin heavy chain, whereas calcification and osteoblast markers, including the osteoblast-promoting factor Runx2, Msx2, ALP, collagen type I, OPN, MMP-2, and PiT-1, are up-regulated [227]. In particular, the activation of PiT-1 increases the intracellular levels of Pi that, in turn, regulates intracellular signaling pathways, such as the Wnt/β -catenin and Axl/growth arrest-specific gene 6 pathways, leading to VC. Despite the similarities between vascular and bone mineralization, it has to be pointed out that, besides the pathologic vs. physiologic consequences, VC is characterized by localized mineralized areas of the elastic component, whereas collagen is rarely involved. Moreover, calcifying VSMCs exhibit reduced viability with increased apoptosis and higher expression of calcification markers (e.g., TNAP or Runx2) although at lower levels compared to bone cells [228].

Calcification increases vessel stiffness and has consequences on hemodynamic properties and on mechanical vascular wall stress. Cyclic stretching of VSMCs further stimulates phenotype switching and adversely affects endothelial function determining impaired NO synthesis, up-regulation of pro-inflammatory and pro-atherogenic factors, increased oxidative stress [227]. To be noted that endothelial dysfunction rests on the repair potential of endothelial progenitor cells and on the ability of these cells to promote the repair of the luminal lining or to acquire a pro-osteogenic phenotype, thus actively contributing to VC [48].

Whereas the arterial intima calcification is generally related to atherosclerotic plaques and ischemic vessel injury, calcification in the vascular media is a frequent end-stage pathological change of chronic diseases [229]. In blood vessels, mineral deposition is mainly localized in necrotic areas within the atherosclerotic plaque or within the elastic lamellae, which are layered between the syncytia of smooth muscle cells. Elastic fibers are primarily composed of elastin molecules assembled with a number of matrix components (i.e., fibrillins, fibulins, glycosaminoglycans/proteoglycans, glycoproteins, lysyl oxidase, lipids as well as calcium-binding proteins), whose amounts and ratios change depending on tissues’ functional requirements, age, and pathologic conditions [14]. Therefore, calcification causes decreased elasticity of vessels and valves and hamper tissue mechanics and blood flow, possibly resulting in heart attack and stroke [230]. ECM organization (e.g., stiffness, porosity, and density) controls cellular differentiation and regulates calcification under the influence of matrix components as collagens [231]. For instance, collagen type I has been shown to promote VSMCs phenotypic differentiation into osteoblast-like cells and, in the course of atherosclerosis, there is an increased amount of collagen type I. Similarly to bones, increased collagen type I content favors HA deposition in the arterial wall, whereas collagen type IV inhibits mineralization [232].

Many reports have highlighted the importance of cell-matrix interactions in transducing signals from the extracellular environment to the nucleus and vice versa; therefore, qualitative and quantitative changes in matrix components modulate cell morphology and behavior [233]. Interestingly, it has been demonstrated in EC, VSMCs, and fibroblasts that mechanical stress (both in the form of cyclic stretch and tensile strain) can modulate the expression of calcification-related genes by up-regulating pro-osteogenic factors such as Sprouty-1, BMP-2, periostin and down-regulating calcification inhibitors as osteopontin and thrombospondin [234]. ECs, in particular, through the endothelial to mesenchymal transition (EndMT), seem to play a key role in initiating VC in the tunica intima through the release of BMP-2 and activation of the Wnt/β-catenin pathway, losing their specific markers (i.e., CD31 and VE-cadherin) and becoming more responsive to BMP/TGF-β signaling pathways [235]. Furthermore, ECs release miRNAs capable of switching VSMCs towards a procalcific phenotype in the tunica media [235]. In vascular tissues, cell plasticity is actively modulated by mechanical stretching that also induces VSMC proliferation as well as migration, apoptosis, phenotypic switching, and vascular remodeling [236]. In these conditions, magnesium can enter VSMCs through the transient receptor potential melastatin 7 channel in response to the up-regulated expression of angiotensin II type I receptor (AT1-R). Since insufficient magnesium has been associated with VC, magnesium levels could be regulated by angiotensin II, thus preventing phosphate-induced calcification [237].

VC is generally associated with age-related diseases, and in vitro aging models have demonstrated an increased susceptibility of cells to pro-osteogenic signals, but recent evidence indicates that the relationship between aging and calcification is likely multifactorial [238]. For instance, senescent VSMCs are characterized by the release of a high number of extracellular vesicles, up-regulation of Runx-2 and TNAP, altered redox balance, mitochondrial dysfunction, and inhibition of Sirtuin-1 with corresponding up-regulation of p53 [238].

To further highlight the complexity of pro-osteogenic signaling mechanisms in vascular tissues, it is worth mentioning the post-transcriptionally regulatory activities exerted by non-coding RNAs as those stored within exosomes [239].

By microarray analysis, during the progression of vascular calcification, it was observed an increased expression of miR-125b, miR-30a, and miR-32 and a decreased expression of miR-29a, miR-210, and miR-320 [240].

For instance, gain- and loss-of-function studies demonstrated that miR-32 up-regulates the expression of BMP-2, RUNX2, OPN, and matrix GLA protein through the PI3K signaling pathway. Consistently, high circulating levels of miR-32 were detected in patients with coronary artery disease (CAD) with calcification compared to patients with CAD in the absence of calcification [240].

The demonstration that in areas of mineral deposition matrix vesicle-like structures can be observed containing high levels of Bax provided evidence that these structures can represent remnants of apoptotic cells [241]. During VC, both ABs and MVs derived from VSMCs promote the accumulation of calcium-phosphate in a microenvironment favoring mineralization. The observation that apoptosis takes place prior to the onset of calcification put forward the hypothesis that apoptosis is a key initiating process for VC [242].

Within this context, oxidative stress up-regulates the expression of RUNX2, leading to the osteogenic conversion of VSMCs. Consistently, Runx2 induces the expression of matrix proteins such as collagen type I, osteocalcin (OC), OPN, bone sialoprotein, and TNAP activity [106,107]. Moreover, mitochondrial ROS promote the expression of tristetraprolin, a RNA destabilizing factor, that reduces the expression of ANKH, an inhibitor of calcification [93].

There are several data indicating that VC is also associated with endoplasmic reticulum stress (ERS)-induced apoptosis and altered calcium homeostasis since the endoplasmic reticulum is the primary site for Ca^2+^ storage [243].

Moreover, in diabetic patients, high glucose interferes with ER function, and ERS generates several pro-apoptotic signals and favors the phenotype transition of VSMCs [244]. Within this context, the antidiabetic drug metformin may counteract vascular calcification reducing osteogenic markers expression (e.g., Runx2 and BMP-2) [245], restoring mitochondrial biogenesis and inhibiting β-glycerophosphate-induced pyruvate dehydrogenase kinase expression, a mitochondrial metabolic regulator associated with mitochondrial dysfunction in calcified VSMCs [246].

Interestingly, it has been recently demonstrated that VC can be reduced by globular adiponectin, a bioactive polypeptide secreted by adipose cells, which negatively correlates with the degree of coronary artery calcification and protects vasculature by inducing NO activation, promoting the expression of osteoprotegerin (OPG) and the repair of EC, while inhibiting the phenotype conversion of VSMC into osteoblast-like cells [247]. These findings may open new perspectives for the treatment of VC in diabetic patients, aiming to reduce cardiovascular complications.

Several studies have shown major sites of VC in atherosclerotic plaques. Atherosclerosis is a pathologic condition characterized by progressive accumulation of lipoproteins within the vessel wall, development of an inflammatory response that alters tissue architecture, and causes reduced blood flow and blood supply to organs. It is one of the leading risk factors for cardiovascular diseases, especially in Western Countries, being detected in 100% of individuals > 65 years of age [248]. It is now well recognized that in atherosclerosis, there are many types of cell death, including necrosis, apoptosis, and autophagy [223]. In the course of the atherosclerotic process, apoptotic cells are few in early plaques, whereas they increase with the evolution of the lesions. Remnants of apoptotic VSMCs remain in the plaque as membrane vesicles and can act as nucleating structures for plaque microcalcifications and enhanced plaque progression. VC is further promoted by mechanical injury, high levels of NO, oxidized low-density lipoproteins (oxLDL), and pro-inflammatory cytokines (e.g., TNF-α by macrophages and IFN-γ by T lymphocytes) [249].

Pro-inflammatory cytokines, as TNF-α and ILs, are involved in diabetic patients and in diabetes associated VC [250]. In particular, TNF-α favors calcium deposition by VSMCs, up-regulation of BMP, expression of ALP through NF-kβ pathway as well as the Msx2-Wnt osteogenic program [251].

By contrast, IL-24 down-regulates calcification and osteoblast markers including Runx2, ALP, OPN, BMP-2, and the Wnt/β-catenin pathway and, therefore, can efficiently attenuate β-glycerophosphate-induced VSMC calcification, decreasing either Bax/Bcl-2 expression ratio and apoptosis [252].

Similarly, OPG exerts anti-atherosclerotic and anti-calcification effects in in vitro and in animal models [253], although the role of OPG may be context-dependent since it was observed increased in serum of patients with VC [17].

### 3.1. Changes in Matrix Components in the Context of Vascular Apoptosis

Survival of anchorage-dependent cells such as EC [254] and VSMC [255] are secured by cell-to-cell and cell-to-matrix contacts, as demonstrated in in vivo models, where up-regulation of the integrin α_v_β_3_ counteracts cell-death pathways by inhibiting p53 activity, decreasing p21 and Bax expression and activating NF-κB [256]. By contrast, down-regulation or truncation of vascular endothelial (VE)-cadherins induces EC apoptosis and abolishes signals by VEGF-A to Akt kinase and Bcl-2 [257,258]. In vitro studies on VSMC cultured within collagen gels have demonstrated that interactions of these cells with tenascin-C through α_v_β_3_ integrins can change cell shape and epidermal growth factor (EGF)-dependent growth by inducing a clustering of the EGF-receptors and may rescue VSMC from apoptosis [259]. Moreover, exposure of human EC to TNF-α and IFN-γ results in suppression of EC α_v_β_3_ activity leading to decreased cell adhesion and survival [260].

Furthermore, the observation that interfering with β1 integrins can modulate the effect of TGF-β1, and Bcl-2 protein expression highlights the importance of the microenvironment and of cell-matrix interactions on apoptotic signaling pathways [261]. It has been demonstrated that through β_1_ integrins, TGF-β_1_ exerts context-dependent bifunctional effects; being an inducer of endothelial cell apoptosis, whereas it prevents VSMC death. In particular, in endothelial cells, TGF-β_1_ stimulation results in decreased Bcl-2 protein levels. Interestingly, the pro-apoptotic effect of TGF-β_1_ on HUVEC (human umbilical vein endothelial cells) can be counteracted by activation of β_1_ integrins and increased expression of the anti-apoptotic factor Bcl-2 [261].

Moreover, it has been demonstrated that p53 suppression can be induced by extracellular matrix survival signals as those generated by fibronectin through the focal adhesion kinase (FAK), whose phosphorylation leads to p53 inactivation and maintenance of cell survival [262,263]. Furthermore, nonfibrillar or monomeric collagens allow VSMCs to undergo proliferation in response to mitogens in culture, while fibrillar collagens inhibit cell proliferation by up-regulating specific inhibitors of the cell cycle [264]. Therefore, the ECM determines whether cells remain quiescent, survive, or multiply in response to growth factors [248].

In EC apoptosis, it has been demonstrated that caspase-mediated degradation represents an early event leading to the intracellular cleavage of FAK, β-catenin, and plakoglobin and to the shedding of VE-cadherin, and therefore disruption of extracellular survival signals [265]. Interestingly, a link between mechanical stimulation and cell survival or death has been suggested by several groups. Indeed, HUVEC cultured under static conditions undergo a basal level of apoptosis, whereas in vitro or in vivo exposure of cells to flow inhibits the apoptotic process [266,267]. These effects involve the shear-induced phosphorylation of Akt/PKB pathway, phosphorylation of the endothelial NO synthase, production of NO that, in turn, inhibits caspase-3 activation and prevents EC apoptosis [268,269,270]. Interestingly, shear stress-dependent up-regulation of Cu/Zn SOD and NO synthase can prevent caspase activation upon a number of apoptotic stimuli (e.g., oxygen free radicals, oxLDL, or TNF-α) [271,272].

Since caspases play an essential role in apoptosis, inhibition of these proteases has been investigated as an approach to reduce apoptotic cell death and to stabilize atherosclerotic plaques, as suggested by treating VSMC with the caspase inhibitor zVAD-fmk [73].

In addition to caspases, atherosclerotic plaques are characterized by overexpression of MMPs, which contribute to matrix degradation, cell detachment, and induction of cell death [273]. MMPs could also modulate apoptosis by cleaving death ligands (e.g., TNF-α and Fas-L) and their receptors, which trigger apoptosis by acting in an autocrine or paracrine manner [274]. For instance, MMP-1, -2, -8, -9, -13 and the MT-MMPs, MMPs 14–17 can all cleave pro-TNF-α, although only ADAM-17/TNF-α converting enzyme (TACE) and, with a lesser specificity, MMP-7 produce the correctly processed soluble form of TNF-α [275].

### 3.2. Death Receptors, Cytokines and Growth Factors in the Context of Vascular Apoptosis

Apoptosis of vascular cells is observed in vivo in normal vessel development and in a number of vascular pathologies. Cell death in atherosclerosis has been firstly reported by Virchow during a lecture given at the University of Berlin in 1858, although, at that time, the term cell death was used to indicate necrosis, a process closely related to chronic inflammation. The observation that cell death in VSMC is due to deregulated expression of c-*myc* and that this process can be reversed by expression of Bcl-2 or by other survival factors such as insulin growth factor (IGF)-1 and platelet-derived growth factor (PDGF) provided the demonstration that programmed cell death may occur in atherosclerosis [276]. It is now well recognized that in atherosclerosis, there are many types of cell death: necrosis, apoptosis, and autophagy [223].

Increased EC apoptosis has been observed in the early phases of atherosclerosis, whereas apoptosis of VSMCs and of macrophages is typically localized in ‘vulnerable’ lesions, which are more prone to rupture. VSMC apoptosis promotes plaque thrombogenicity by exposing phosphatidylserine on the surface of apoptotic cells that can act as a substrate for thrombin generation and activation of the coagulation cascade.

In agreement with these observations, the death receptor Fas is expressed throughout the vessel wall, and the Fas receptor/Fas-L pathway, which is involved in cell apoptosis, has been widely recognized in vascular diseases [277].

Interestingly, monocyte/macrophages induce human VSMC apoptosis through Fas-L/Fas, NO, and TNF-α also acting in synergy with inflammatory cytokines as IL-1β and IFN-γ [278,279]. Whereas TNF-α up-regulates macrophage iNOS and surface Fas-L through autocrine regulation of TNF-R1 and TNF-R2, Fas and TNF-R1 initiate caspase-dependent death signaling by similar mechanisms. Interestingly, although TNF inhibition hinders Fas-L, the opposite is not true since Fas-L inhibition does not hamper TNF-α surface expression, indicating that Fas-L is downstream of TNF-α. In contrast, NO and TNF-α appear to be mutually dependent because inhibiting NO reduces TNF-α and vice versa [278].

Lindner and co-workers [280] demonstrated that preactivated peripheral blood mononuclear monocytes could induce EC apoptosis through a mechanism that is, at least in part, dependent on TNF-α, since it can be efficiently inhibited by anti-TNF-α monoclonal antibodies or by treatment with IL-10, an anti-inflammatory cytokine with anti-apoptotic activity. It has to be mentioned that TNF-α, similarly to other inflammatory cytokines, induces the Bcl-2 homolog human A1, but can also activate the NF-κB pathway, thus indicating that TNF-α can initiate both pro-apoptotic and anti-apoptotic pathways in a context-dependent mode [281,282].

In the course of endothelial inflammation, it is worth mentioning that the anti-apoptotic proteins Bcl-2 and Bcl-XL are able to down-regulate EC activation through NF-κB inhibition. Bcl-2 and Bcl-XL may, therefore, be cytoprotective, counteracting both pro-apoptotic and pro-inflammatory stimuli [283].

Tumour necrosis factor-related apoptosis-inducing ligand (TRAIL), a member of the TNF protein superfamily, induces apoptosis, binding to death signaling receptors DR4 and DR5 [284]. Although it was suggested that TRAIL-induced apoptosis is typically triggered in transformed cells, a number of studies provided evidence that TRAIL is highly expressed in atherosclerotic lesions involving endothelial and smooth muscle cells [285]. These events may accelerate the switch of a stable plaque to a rupture-prone plaque [286]. Interestingly, TRAIL-induced apoptosis can be inhibited by OPG, a soluble decoy receptor also acting as regulator of osteoclastogenesis preventing RANKL–RANK binding and bone resorption [287].

Among growth factors, fibroblast growth factor 21 (FGF21) is considered a mitokine exerting several metabolic functions possibly regulating autophagy and cell death processes [288]. Moreover, it has been demonstrated that FGF21 can ameliorate atherosclerosis inhibiting C/EBP homologous protein (CHOP) and caspase-12 signaling pathways associated with ERS-mediated apoptosis [289,290]. CHOP is a transcription factor that regulates the expression of numerous pro-apoptotic proteins leading to oxidative stress and apoptosis, is also up-regulated in calcified aortas [289]. Caspase-12 is a cysteine protease that is specifically activated by ERS, and subsequently, it activates caspase-9, caspase-3, and apoptosis, and is involved in VC [291]. The observation that both CHOP expression and caspase-12 can be significantly reduced by FGF21 further demonstrates that the growth factor may reduce ERS, apoptosis, and VC [289,292].

### 3.3. Mitochondria and Reactive Oxygen and Nitrogen Species in the Context of Vascular Apoptosis

Calcified blood vessels are associated with mitochondrial damage and dysfunction, mainly affecting the mitochondrial electron transport chain and, consequently, ROS production that, in turn, regulates cell proliferation, apoptosis, and Ca^2+^ storage [75]. Mitochondrial dysfunction or abnormalities lead to the loss of mitochondrial membrane potential, enhanced intracellular ROS generation, Ca^2+^ overload, and decreased ATP synthesis. Moreover, mitochondria accumulate calcium in an energy-dependent manner, and excessive intake of Ca^2+^ by mitochondria triggers the opening of permeability switching pores and the release of cytochrome c into the matrix, which led to apoptosis and to VC [75].

Atherosclerotic plaques develop as a consequence of the accumulation of circulating lipids and the subsequent migration of inflammatory cells. This process is markedly enhanced by oxLDL that have been shown to exert cytotoxic effects on cultured EC. In vitro studies have demonstrated increased apoptotic cell death of aortic EC exposed to cholesterol oxides or to oxLDL [293]. OxLDL promotes apoptosis through the Fas signaling pathway [294], down-regulating Bcl-2 and activating caspase-3 [295], but cell death can be prevented by chelating extracellular calcium or by inhibiting calcium influx, indicating that increase of cytosolic calcium precedes apoptotic events [296].

In VSMC, the pro-apoptotic effects of pro-inflammatory cytokines (TNF-α, IFN-γ, and IL-1) are also associated with high production of NO via iNOS [297]. This process is mediated by TNFα and FasL interactions with their receptors [298]. By contrast, the anti-inflammatory cytokine IL-10 is associated with decreased levels of iNOS expression and apoptotic cell death [299]. These findings suggest that apoptosis results from an excessive inflammatory reaction. Moreover, in VSMCs, NO-induced apoptosis is prevented by inhibiting the cGMP-dependent protein kinase Iα, or by adding angiotensin II [300]. It is noteworthy that NO, at physiological concentrations, acts as an anti-apoptotic factor, whereas it exerts a pro-apoptotic effect at high levels (e.g., during inflammatory response) [301,302]. Similarly, angiotensin II may have dual effects on VSMC apoptosis, preventing or promoting apoptosis through angiotensin II type 1 or type 2 receptor stimulation, respectively [303].

### 3.4. Microribonucleic Acids and Long Non-Coding RNA in the Context of Vascular Apoptosis

Several miRs have been reported to control VSMC turnover and apoptosis targeting growth factor pathway intermediates, ROS production, transcription factors, cell cycle, and/or apoptosis control points. For instance, miR-21, miR-26a, miR-29b, and miR-126 have been described in VSMC as regulators of the ratio between apoptosis and proliferation, whereas miR-143 and miR-145 modulate the growth process by phenotype changes [304].

Furthermore, miR-25 can inhibit corticosterone-induced VSMC apoptosis by targeting the pro-apoptotic protein MOAP1 and possibly also the p70S6k pathway [305]. MOAP1 promotes caspase-dependent apoptosis by binding pro-apoptotic BAX via its Bcl-2 homology-3- (BH3-) like domain, and it is up-regulated upon several apoptotic stimuli [306]. Since MOAP is a direct target for miR-25, it has been suggested that miR-25-dependent down-regulation of MOAP1 may represent a key mechanism in apoptosis inhibition with positive effects on atherogenesis and eventually on calcification [305]. It has to be further investigated if these data may also be relevant for therapeutic implications in humans.

miR-148b has been reported as an inhibitor of atherosclerosis by decreasing VSMC proliferation and migration, and increasing apoptosis [307].

Interestingly, in addition to miR, recent studies in calcified vessels and valves provided evidence that lncRNAs (HOTAIR, H19, TUG1, and ES3) regulate a number of signaling pathways, such as Wnt/β catenin and NOTCH, or target specific miR (e.g., miR-34c, miR-125, miR-204, and miR483). For instance, the taurine up-regulated gene 1 (TUG1) is a functional lncRNA promoting macrophage’s growth and the atherosclerotic inflammatory response regulating the expression of miR-133a that, in turn, modulates VSMC proliferation and endothelial cell apoptosis [308]. Moreover, TUG1 is overexpressed when cells are exposed to oxLDL and can increase IGF2 expression by competitively sponging miR-148b. Therefore, it has been suggested that TUG1 regulates proliferation and apoptosis of VSMC and HUVEC by miR-148b/IGF2 axis, thus providing a novel patho-mechanism for atherosclerosis [309].

Apoptosis in EC is suppressed by lncRNA HOTAIR, and these effects may contribute to control cell proliferation and migration in the course of atherosclerosis [310].

## 4. Conclusions

In summary, aberrant mineralization of soft connective tissues is a complex multifactorial process modulated either at pre- and post-transcriptional levels, and it has been observed to progressively increase its frequency due to longer life expectancy and to the higher incidence of chronic degenerative diseases. Ectopic calcification negatively affects the quality of life of aged patients and is responsible for severe complications, mainly affecting joints and the cardiovascular system. In most cases, extra-skeletal mineralization has been observed to be the consequence of apoptotic cell death or at least to intersect some apoptotic signaling pathways. Therefore, apoptosis plays an active role in the calcification process, and ABs may serve as nucleation sites for the crystal deposition as observed in cartilage and in vascular mineralization. Moreover, numerous studies, performed both in vitro and in vivo, have highlighted as extraosseous calcification shows similarities to physiological skeletal mineralization. However, factors regulating apoptosis and/or calcification exert multiple effects dependent, for instance, on cell type, on cell-cell and on cell-matrix interactions, on extracellular matrix composition, and on mechanical forces. The frequent occurrence of dual effects is, therefore, cell and context-dependent, as clearly shown in the two models we have analyzed in the present review (i.e., chondrocytes in the cartilage tissue and EC and VSMC in vascular tissue).

At present, there are a number of drugs (e.g., metformin, statins, bisphosphonates, and anti-oxidants) which may exert their effects interfering with cell fate and which have also been associated with hydroxyapatite deposition; however, therapeutic approaches capable of inhibiting ectopic calcification without negatively interfering on the whole mineralization process are in their infancy. Therefore, better exploring the relationships between apoptosis and hydroxyapatite deposition will be highly relevant to identify new and more effective therapeutic treatments against extraosseous calcification.

## Figures and Tables

**Table 1:** Type of crystals found in extraosseous calcification.

Mineral	Short Name	Chemical Formula	Mineralization Site	References
Amorphous calcium phosphate	APC	Ca_9_(PO_4_)_6_	Atherosclerotic plaque, aortic valves, and brain	[21,22,23]
Brushite or dicalcium phosphate dihydrate	DCPD	CaHPO_4_·2(H_2_O)	Heart valves and kidney stones	[24,25]
Calcium carbonate	CaCO	Ca_10_(PO_4_)_6_(OH)_2−2x_(CO_3_)_x_ or Ca_10−x_(PO_4_)_6−x_(CO_3_)_x_(OH)_2−x_	Tendons	[26]
Calcium oxalate	CaOx	CaC_2_O_4_	Breast, renal stones, and vascular	[27,28]
Calcium pyrophosphate dihydrate	CPPD	Ca_2_P_2_O_7_	Knee joint, meniscus, and tendons	[29,30,31,32]
Hydroxyapatite	HA	(Ca_10_(PO_4_)_6_OH_2_)	Aortic valve, brain, cardiovascular tissue, kidney, skin, and tendons	[23,33,34,35,36,37]
Tricalcium phosphate	TCMP	Ca_3_(PO_4_)_2_	Breast and tendons	[26,38]
Octacalcium phosphate	OCP	Ca_8_(HPO_4_)_2_(PO_4_)_4_·5H_2_O	Heart valves, knee joint compartments	[24,39,40]
Whitlockite	WH	Ca_18_Mg_2_(HPO_4_)_2_(PO_4_)_12_	aorta, breast, cartilage, prostate, salivary glands	[41,42,43,44,45,46]

## Data Availability

Not applicable.
